# THERPA v2: an update of a small molecule database related to prion protein regulation and prion disease progression

**DOI:** 10.1080/19336896.2019.1689789

**Published:** 2019-11-22

**Authors:** Sol Moe Lee, Sung Soon Kim, Heebal Kim, Su Yeon Kim

**Affiliations:** a Division of Bacterial Disease Research, Center for Infectious Diseases Research, Korea National Institute of Health, Centers for Disease Control & Prevention, Cheongju-si, South Korea; b Department of Agricultural Biotechnology and Research Institute of Agriculture and Life Sciences, Seoul National University, Seoul, South Korea

**Figure 1.**
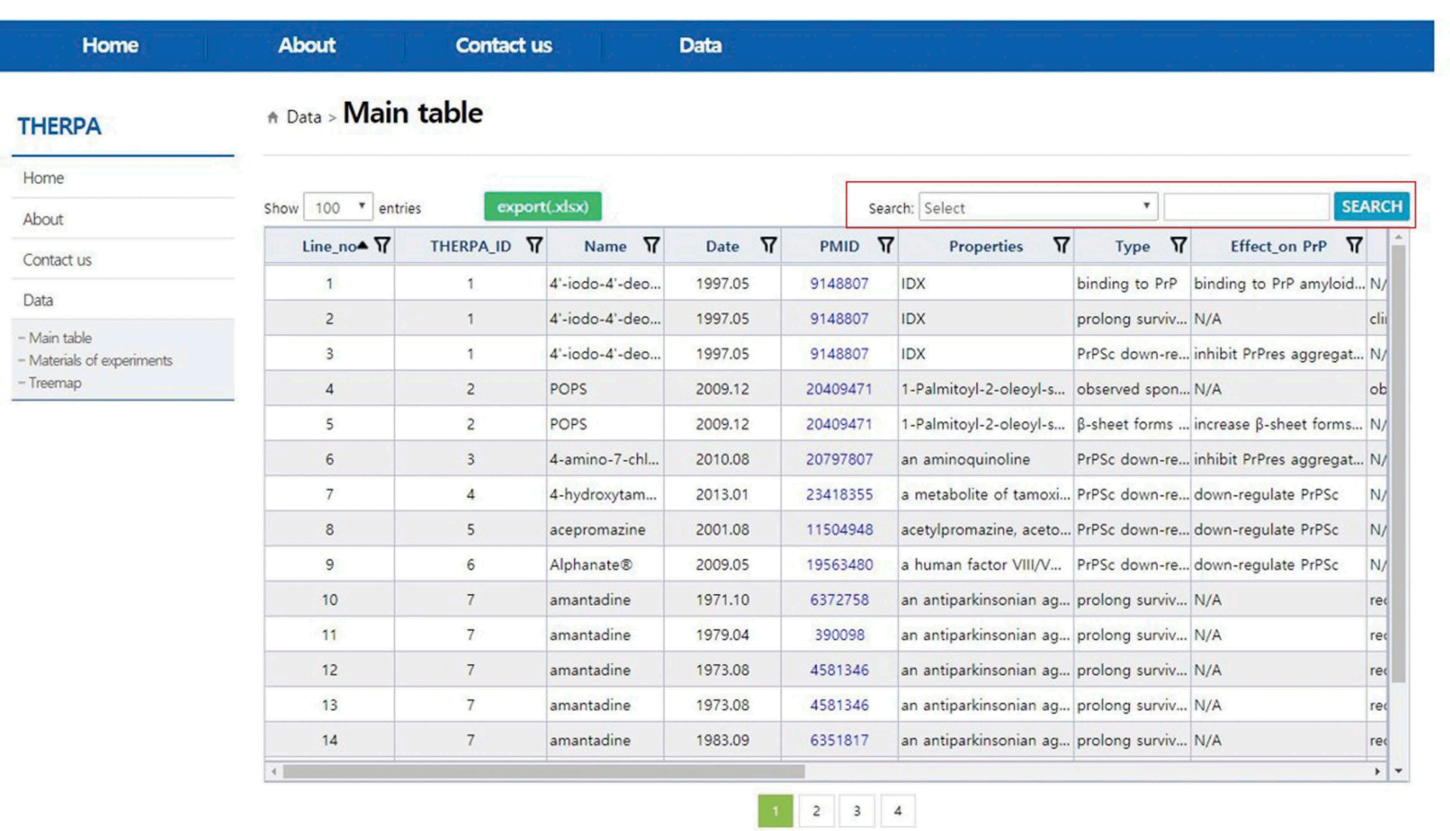
Snapshot of the updated *Main table*. Detailed information for the 144 small molecules is listed and classified into 14 categories. Red square in the upper right denotes a field for search.

Prion diseases are rare, rapidly progressive neurodegenerative disorders that affect mammalian species [1,2]. Abnormal accumulation of infectious form of the prion protein in the brain causes prion disease. Various small molecules have been used to inhibit and treat this disease [3,4]. We built a repository of therapeutic molecules associated with prion protein and prion diseases (THERPA) to allow users to easily browse information describing various small molecules in publicly available articles [5]. THERPA is an open-access database containing data regarding small molecules related to prion protein and prion diseases, which is aimed at allowing researchers to easily explore and analyse data of interest. Here, we describe the relocation of the webpage and THERPA database updates. The THERPA repository has been relocated to the Korea National Institute of Health website for stable maintenance (www.nih.go.kr/therpa). The e-Government Standard Framework (www.egovframe.go.kr) was used to create the current website, which is compatible with the mobile web environment. THERPA has been expanded to cover 144 small molecules and their 353 relationships. A table template in the *Main table* in the current version was created using SBGRID (https://sbgrid.co.kr). To perform a search, users can specify a category by selecting one search category and entering a search text or keyword, after which the users click on the SEARCH button to execute the search (Figure 1). Users can download EXCEL files by clicking on the green button labelled, ‘export (.xlsx)’ at the upper left of the table. The *materials for experiments* and *treemap* pages were also updated followed by additionally curated small molecules and their relations. The manually curated THERPA will be updated regularly with new datasets to provide more valuable resources regarding small molecules and their role in prion protein regulation and in managing prion diseases. The repository would facilitate meta-analysis and would be useful for understanding disease mechanisms and developing therapeutic strategies.
